# Laxative Effects of a Standardized Extract of *Dendropanax morbiferus* H. Léveille Leaves on Experimental Constipation in Rats

**DOI:** 10.3390/medicina57111147

**Published:** 2021-10-22

**Authors:** Ju-Ryun Na, Ki Hoon Lee, Eun Kim, Kwontack Hwang, Chang-Su Na, Sunoh Kim

**Affiliations:** 1 Central R&D Center, B&Tech Co., Ltd., Gwangju 61239, Korea; ryun1225@daum.net (J.-R.N.); leekh3261@daum.net (K.H.L.); rubsang84@gmail.com (E.K.); 2 Department of Food Science and Nutrition, Nambu University, Gwangju 62271, Korea; hwangskt@gmail.com; 3 College of Korean Medicine, Dongshin University, Naju-si 58245, Korea; nakugi@daum.net

**Keywords:** *Dendropanax morbiferus* H. Lév., constipation, laxative effect, low-fiber diet, loperamide

## Abstract

*Background and Objectives:* This study aimed at investigating the laxative effects of a standardized aqueous extract of *Dendropanax morbiferus* H. Lév. on two different constipation rat models. *Materials and Methods:* Animal studies were conducted with low-fiber diet-induced and loperamide-induced constipation animal models, and isolated colons were used in ex vivo analysis to determine the changes in colonic motility caused by *D. morbiferus* H. Lév. leaf extract (DPL). *Results:* The results showed that DPL administration significantly improved certain reduced fecal parameters (number, weight, and water content of the stools) in a both low-fiber diet and loperamide-induced constipation models without adverse effects of diarrhea. The laxative effect of DPL was confirmed to improve the charcoal excretion time upon DPL treatment in a low-fiber diet or loperamide-induced constipation model through gastrointestinal (GI) motility evaluation using the charcoal meal test. In addition, when DPL was administered to RAW264.7 cells and loperamide-induced constipation model rats, the production of prostaglandin E_2_ (PGE_2_) increased significantly in cells and tissue. Furthermore, DPL dose-dependently stimulated the spontaneous contractile amplitude and frequency of the isolated rat colon. *Conclusion:* Although our study did not provide information on the acute or chronic toxicity of DPL, our results demonstrated that DPL can effectively promote defecation frequency and rat colon contraction, providing scientific evidence to support the use of DPL as a therapeutic application. However, further toxicity studies of DPL are needed prior to the initiation of clinical trials and clinical applications.

## 1. Introduction

Constipation is defined as a gastrointestinal disorder characterized by difficult, irregular, or deficient defecation [1]. Diagnostic criteria of functional constipation are based on the following Rome III criteria [2]: (I) two or more of the following occurrences > 25% of the time: straining, hard stools, sensation of incomplete evacuation, sensation of anorectal obstruction/blockage, manual maneuvers to facilitate evacuation, or fewer than three defecations/week; (II) loose stools are rarely present without laxatives; and (III) insufficient criteria for irritable bowel syndrome. Generally, stool softeners, osmotic agents, bulking agents, and stimulant laxatives are used to treat constipation [3]. However, laxatives can cause cardiac adverse effects and artery contraction [4,5,6].

*Dendropanax morbiferus* H. Lév., also called *Hwangchil*, has been used traditionally to treat intestinal disorders in Korea [7]. Active compounds in *D. morbiferus* H. Lév. leaf extract (DPL) have various pharmacological properties [8,9,10]. Previously, we proposed the use of quercetin and kaempferol as chemical markers for the quality control of *D. morbiferus* after analyzing parts, seasons, and extraction methods [11]. Moreover, we reported the antiobesity effect [12] and antihypertensive effect [13,14] of DPL. However, study about the laxative effect of DPL is still largely unknown. Therefore, in this study, the laxative effects of DPL were investigated in two different animal models of constipation, and the detailed mechanisms were explored. Hence, the aim of the present study was to confirm the preventive and therapeutic effects of DPL on low-fiber diet- and loperamide-induced constipation rat models in vivo and the modulatory effects of DPL on the contraction of ex vivo colonic smooth muscle in rats.

## 2. Materials and Methods

### 2.1. Reagents

DMEM (Lonza, Basel, Switzerland), fetal bovine serum (FBS, Invitrogen, Inc., Grand Island, NY, USA), and penicillin/streptomycin (Invitrogen, Inc.) were used for the cell culture.

All other chemicals were purchased from Sigma-Aldrich (St. Louis, MO, USA).

### 2.2. Preparation of Extracts

In order to ensure the standardization of DPL and reproducibility of efficacy, *Dendropanax morbiferus* H. Lév., an evergreen broad-leaved tree, harvested by selecting the region (34°35′20.4″ N, 126°48′04.2″ E, Gangjin-gun, Jeollanamdo, Korea) and season (December), was used in this study. The leaves were dried in the hot air oven at 55 °C for 24 h. For the aqueous leaf extract, 100 g of the leaves were boiled in 2000 mL distilled water (1:20, *w*/*v*) at 100 °C for 4 h and filtered through a filter paper (Whatman No. 1), then freeze-drying the extract [13,14]. Normally, 27 g extract was obtained from 100 g of dried leaves of *D. morbiferus*. These steps resulted in the sample labeled DPL, and the sample was stored at 10 °C before use in the experiment.

### 2.3. Solvent-Solvent Fractionation and HPLC Analysis of Extracts

DPL was fractionated to *n*-hexane, chloroform (CHCl_3_), ethyl acetate (EtOAc), *n*-butanol (*n*-BuOH), and water fractions (Supplemental Figure S1). The amounts of chlorogenic acid, cryptochlorogenic acid, neochlorogenic acid, quercetin, and kaempferol in DPL were compared with the high-performance liquid chromatography (HPLC) method according to our previously reported standard method [11,15]. The samples were dissolved in 50% methanol, sonicated for 20 min, and then analyzed using an Agilent 1260 HPLC system (Agilent Technologies, Palo Alto, CA, USA) equipped with an Eclipse XDB-C_18_ column (4.6 × 250 mm, 5 μm). The detection wavelength was set to 370 nm. Quantitative HPLC analysis was replicated three times.

### 2.4. Cell Culture and Prostaglandin E_2_ (PGE_2_) Content Measurement

Murine macrophage RAW264.7 cells were purchased from Korea Cell Line Bank (KCLB 40071, Seoul, Korea) and were grown in DMEM supplemented with 10% FBS at 37 °C in a humidified atmosphere under 5% CO_2_. The cells were grown until 70% confluence and then incubated with DPL for 30 min. The PGE_2_ extraction and analysis were performed using a specific enzyme immunoassay (EIA) kit (Cayman, Ann Arbor, MI, USA) according to the manufacturer’s instructions.

### 2.5. Animals and Grouping

**Figure 1:**
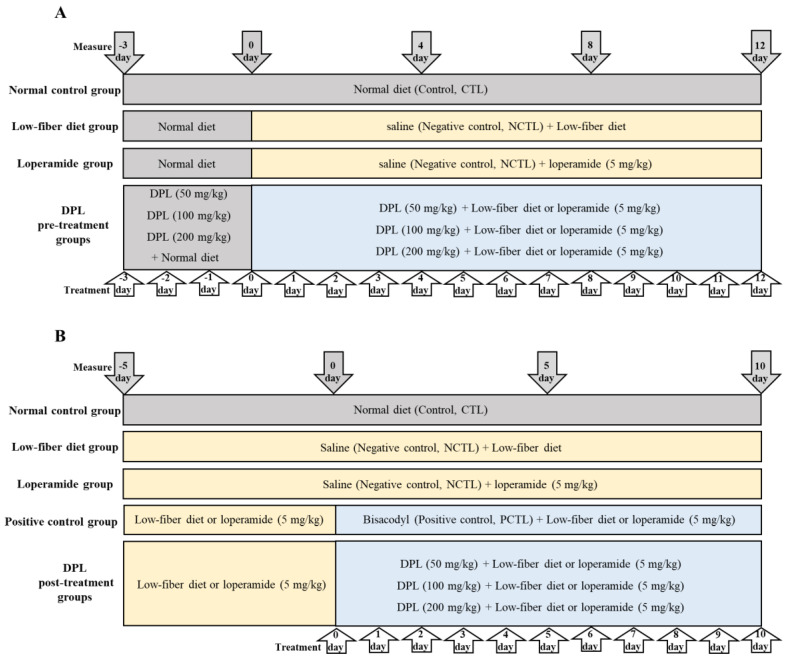
The scheme of the *D. morbiferus* H. Lév. leaf extract (DPL) (50, 100, and 200 mg/kg) laxative experiment. The extracts and physiological saline solution were administered once a day during the experiment. (**A**) DPL was pretreated for three days before administration of a low-fiber diet or loperamide, followed by administration for twelve days. (**B**) The therapeutic effect of constipation by post-treatment with DPL. After five days of low-fiber diet or loperamide administration, three different doses of DPL and bisacodyl (0.25 mg/kg), as a positive control (PCTL), were administered.

Sprague Dawley (SD) rats weighing 180 to 200 g were provided by Central Lab Animal, Inc. (Seoul, Korea). The experiment was conducted according to the international guidelines [16]. In this study, 7 animals per group were employed to minimize the number of animals used. The animal groups to verify the preventive effect of DPL in both the low-fiber diet- and loperamide-induced constipation model were divided randomly into the following 5 groups of equal numbers (*n* = 7), avoiding intergroup differences in body weight: the control group receiving saline and fed a regular diet; the low-fiber diet-induced constipation group or the loperamide-induced constipation group (negative control, NCTL); treatment groups 50, 100, and 200 mg/kg DPL, and fed a low-fiber diet; or treatment groups 50, 100 and 200 mg/kg DPL, fed a normal and receiving loperamide (Figure 1A). The animal groups to verify the therapeutic effect of DPL in both the low-fiber diet and loperamide-induced constipation model were divided randomly into the following 6 groups of equal numbers (*n* = 7), avoiding intergroup differences in body weight: the control group receiving saline and fed a regular diet; the low-fiber diet-induced constipation group or loperamide-induced constipation group (negative control, NCTL); the bisacodyl-treated group fed a low-fiber diet or the bisacodyl-treated group fed a normal diet and receiving loperamide (positive control, PCTL). Treatment groups had 50, 100 and 200 mg/kg DPL, and were fed a low-fiber diet, or had 50, 100 and 200 mg/kg DPL, and were fed a normal diet and receiving loperamide (Figure 1B). DPL (50, 100, and 200 mg/kg) at 1 mL/rat, dissolved in physiological saline solution, was orally administered using a stainless oral sonde (Jungdo-BNP, Seoul, Korea), and the CTL and NCTL rats received the same volume of saline solution in a same method. In total, 180 male rats were used for in vivo and ex vivo studies. All experimental protocols were approved by the institutional animal care and use committee (IACUC) of B&Tech Co., Ltd., Korea (Approval number: BT-008-2020, 9 July 2020).

### 2.6. Induction of Low-Fiber Diet-Induced Constipation

In this study, we applied two methods to test the ability of DPL to prevent and alleviate constipation (Figure 1A,B). The experimental groups (*n* = 7/group) were designed as a control group (consumed a regular diet; Purina, Inc., Seoul, Korea) and an induced constipation group (fed a low-fiber diet, Supplemental Table S1, Samtako Bio Korea, Inc., Osan, Korea) [17,18]. Specifically, to test the preventive effects of DPL (50, 100, and 200 mg/kg), the samples were treated once a day for 15 days starting three days before feeding on a low-fiber diet. After pretreatment with DPL for three days, DPL was administered once daily during the low-fiber diet administration period.

To measure the therapeutic effects of DPL (50, 100, and 200 mg/kg) or bisacodyl (0.25 mg/kg), a low-fiber diet was fed for five days and then samples were treated with a low-fiber diet for ten days. DPL-treated groups were administrated with DPL dissolved in saline for each concentration once a day during the experiment. The normal control group (NCTL) was orally administrated the same amount of saline as a vehicle once a day during the experiment. The positive control group (PCTL) was orally administrated with bisacodyl dissolved in saline once a day during the experiment.

All animals had free access to food and water during the entire study period. When the experimental period was complete, animals were anesthetized by intraperitoneal injection (i.p.) of 80 mg/kg pentobarbital sodium (Sigma-Aldrich, St. Louis, MO, USA), followed by decapitation.

### 2.7. Induction of Loperamide-Induced Constipation

In this study, we carried out two experiments to validate the ability of DPL to prevent and alleviate constipation (Figure 1A,B). The experimental groups (*n* = 7/group) were designed as a control group (consumed a regular diet; Purina, Inc. Korea) and a constipation group in which constipation was induced by loperamide [16,17,18,19,20,21,22,23]. To test the ability of DPL (50, 100, and 200 mg/kg) to prevent constipation, the samples were treated once a day for 15 days starting three days before administering loperamide. After pretreatment with DPL for three days, DPL was administered once daily during the loperamide administration period. DPL (50, 100, and 200 mg/kg) was dissolved in saline and administered orally 1 h after oral administration of 5 mg/kg of loperamide, daily for twelve days.

To measure the therapeutic effects of DPL (50, 100, and 200 mg/kg) or bisacodyl (0.25 mg/kg), loperamide was fed for five days and then samples were treated with loperamide for ten days. Constipation was induced in rats through the oral administration of 5 mg/kg of loperamide, once a day for ten continuous days at 1 h before administration of DPL. The positive control group (PCTL) was orally administrated with bisacodyl dissolved in saline once a day during the experiment.

All animals had free access to food and water during the entire study period. When the experimental period was complete, animals were anesthetized with 80 mg/kg pentobarbital sodium (i.p.), followed by decapitation.

### 2.8. Measurement of Fecal Parameters

Age (weeks), body weight (g), daily food intake (g), daily water intake (mL), and mass of feces (g) were recorded daily at 9:00 a.m. The total number of feces and total weight of the feces were assessed for each rat for 1 day. The stool water content (%) is calculated as follows: stool water content (%) = [(feces weight before dried − feces weight after dried)/feces weight before dried] × 100.

### 2.9. Gastrointestinal (GI) Motility Test

The charcoal meal excretion test was performed on the last day of the experiment to assess GI motility. Each rat was fed 1 mL of charcoal meal (3% suspension of activated charcoal in 0.5% aqueous methylcellulose) as previously described [18]. Briefly, charcoal meal was orally treated 1 h after sample administration, and the number of black stools in each rat was measured at 2 h intervals for a total of 24 h.

### 2.10. Measurement of PGE_2_ Level in Colons

PGE_2_ extraction and analysis in the rat colon were performed using PGE_2_ assay kit (Cayman Chemical, Ann Arbor, MI, USA). Briefly, the rat colon was snap-frozen in liquid nitrogen. Frozen tissue was pulverized to fine powder under dry ice to extract PGE_2_. The frozen tissue powder (200 mg) was homogenized in 1 mL of PBS (containing 1 mM EDTA; pH 7.4) on ice using an ultrasonic processor. After complete lysis of samples, the supernatant was measured by the method of the manufacturer’s instructions.

### 2.11. Ex Vivo Measurement of Contractile Activity in Isolated Segments of Rat Colon

SD rats (weighing 160–200 g) were anesthetized and sacrificed by cervical dislocation after fasting for 12 h. After removal of stools inside the colon, a 2 cm long colonic segment was placed immediately in an organ bath with Krebs solution bubbled with 5% CO_2_ and 95% O_2_. The contractile activity of the rat colon was measured according to the same method as in our former study [18]. Briefly, the colon tissue was stabilized in an organ chamber for 1 h with a resting tension of 2 g with Krebs solution at 37 °C using a constant-temperature circulator. Contractions were recorded using a force-displacement transducer (AD Instruments, Castle Hill, NSW, Australia) under a basal tension of 0.5 g. The amplitude of contractions was calculated as the average over 1 min. The frequency of contractions was measured over 5 min.

### 2.12. Statistical Analysis

The results are presented as the mean and standard deviation (SD) from three independent experiments. The data were analyzed by Student’s *t*-test or two-way analysis of variance (ANOVA) with GraphPad Prism version 8.0.0 for Windows (GraphPad, Inc., San Diego, CA, USA) software programs. Differences at the *p* < 0.05 level were considered statistically significant.

## 3. Results

### 3.1. HPLC Analysis of DPL

**Figure 2:**
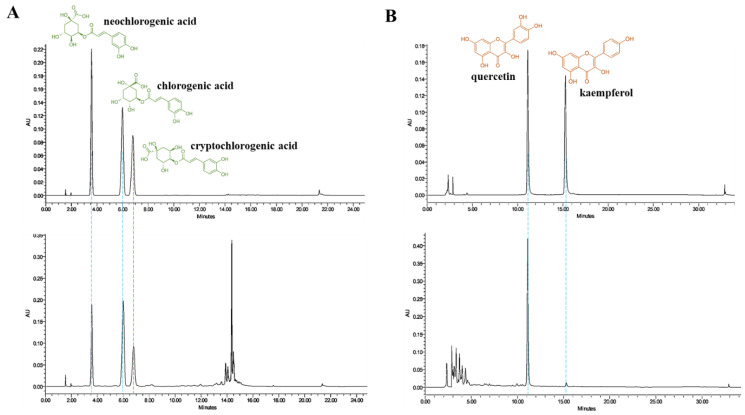
The high-performance liquid chromatography (HPLC) profiles of *D. morbiferus* H. Lév. leaf extract (DPL). (**A**) Column: XDB-C_18_; solvent system for neochlorogenic acid, chlorogenic acid, and cryptochlorogenic acid, a gradient system from 10:90 to 0:100 of acetonitrile with phosphoric acid, 0–25 min was used. (**B**) Column: PhenoSphere™ 5 µm ODS (2); solvent system for quercetin and kaempferol, a gradient system with trifluoroacetic acid/water with acetonitrile as follows: 0–2 min, 80:20; 2–25 min 55:45; 25–30 min, 0:100; 30–31 min, 80:20. The flow rate was 1 mL/min, and for detection, a diode-array detector (DAD) at 370 nm was used. Quantitative HPLC analysis was replicated three times.

The characterization and identification of natural compounds in DPL were performed by HPLC. The three organic acids and two flavonoids in DPL were tentatively identified in accordance with retention times. As shown in Figure 2A, the identified organic acids were neochlorogenic acid (19.24 ± 0.13 mg/g), chlorogenic acid (33.11 ± 0.16 mg/g), and cryptochlorogenic acid (23.41 ± 0.14 mg/g). As shown in Figure 2B, the identified flavonoids were quercetin (14.03 ± 0.11 mg/g) and kaempferol (0.60 ± 0.04 mg/g).

### 3.2. Effect of DPL Administration on Feeding Behavior in Rats with Low-Fiber Diet-Induced Constipation

To evaluate the effect of DPL on feeding behavior of constipated rats, we monitored for feeding behavior in rats with low-fiber diet-induced constipation. Body weight, food intake, and water intake did not differ significantly between the control group (CTL) and the low-fiber diet-induced constipation group (NCTL) during the experiment (Supplemental Table S2). Furthermore, no toxicological revelation on feeding behavior was detected at any of the tested doses of DPL. Taken together, these results show that a low-fiber diet and DPL administration did not induce alterations in feeding behavior.

### 3.3. Preventive Effects of DPL Pretreatment on Low-Fiber Diet-Induced Constipation

**Figure 3:**
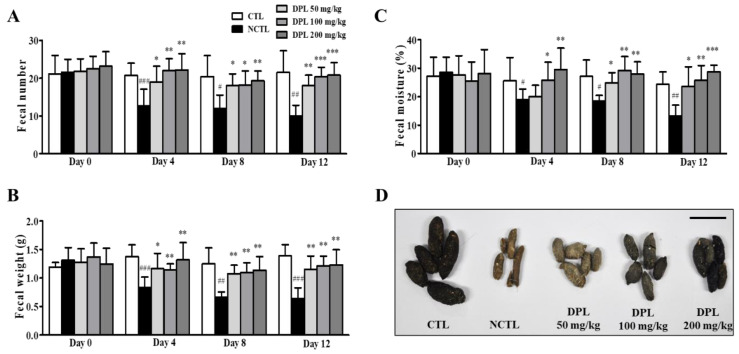
The preventive effects of *D. morbiferus* H. Lév. leaf extract (DPL) on low-fiber diet-induced constipation. At 0, 4, 8, and 12 days, the total number (**A**), weight (**B**), and water content (**C**) of stools were measured. The fecal water content was calculated using the fresh and dry weights of stools. (**D**) Stool morphological characteristics. At 12 days, digital camera images of the stools were taken after collection. Scale bar represents 1 cm. Stools were collected from seven rats per group, and each parameter was assayed in triplicate. Each bar represents the mean ± SD for seven rats. ^#^ Significant difference at *p* < 0.05, ^##^ at *p* < 0.01, and ^###^ at *p* < 0.001 compared with the control group (CTL). * Significant difference at *p* < 0.05, ** at *p* < 0.01 and *** at *p* < 0.001 compared with the low-fiber diet-induced constipation group (negative control group; NCTL).

The preventive effects of DPL pretreatment three days before the low-fiber diet administration were evaluated. The results showing the preventive effects of DPL on constipation are shown in Figure 3. Compared with CTL, administration of a low-fiber diet (12 days) caused a significant decrease in the number (*p* < 0.01), weight (*p* < 0.001), and moisture content (*p* < 0.01) of stools. The 50, 100, and 200 mg/kg DPL-treated groups showed significant increases (*p* < 0.01, *p* < 0.001, and *p* < 0.001, respectively) in the number of stools compared with the NCTL at 12 days (Figure 3A). The 50, 100, and 200 mg/kg DPL-treated groups showed significant increases (*p* < 0.01, *p* < 0.01, and *p* < 0.01, respectively) in the weight of stools compared with the NCTL at 12 days (Figure 3B). Interestingly, the 50, 100, and 200 mg/kg DPL treated groups showed significant increases (*p* < 0.05, *p* < 0.01, and *p* < 0.01, respectively) in the number and weight of stools compared with the NCTL at 4 days. Moreover, the 100 and 200 mg/kg DLP-treated groups showed a significant increase (*p* < 0.05 and *p* < 0.01, respectively) in the water content of stools, whereas the 50 mg/kg DPL-treated group did not show any significant changes compared with the NCTL at 4 days (Figure 3C). However, the 50, 100, and 200 mg/kg DPL-treated groups showed significant increases (*p* < 0.05, *p* < 0.01, and *p* < 0.001, respectively) in the water content of stools compared with the NCTL at 12 days. Furthermore, diarrhea was not observed by DPL treatment (Figure 3D).

**Figure 4:**
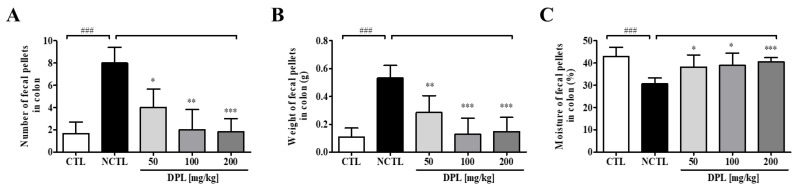
The preventive effects of *D. morbiferus* H. Lév. leaf extract (DPL) on low-fiber diet-induced constipation. The differences in total number (**A**), weight (**B**), and water content (**C**) of stools contained in the intestines at 12 days induced by pretreatment with DPL three days before constipation induced by the low-fiber diet are shown. ^###^ Significant difference at *p* < 0.001 compared with the control group (CTL). * Significant difference at *p* < 0.05, ** at *p* < 0.01 and *** at *p* < 0.001 compared with the low-fiber diet-induced constipation group (negative control group; NCTL).

As shown in Figure 4A,B, compared with the CTL, the NCTL showed significantly increased remnant fecal numbers (*p* < 0.001) and fecal weight (*p* < 0.001) in the colon. However, at sacrifice, the remnant fecal number and fecal weight in the colon were dose-dependently decreased in rats in the DPL treatment groups compared with those in the NCTL. Additionally, we measured the moisture in remnant feces in the colon. We observed that the moisture in the NCTL was significantly decreased (*p* < 0.001) compared with that in the CTL; however, the moisture in the DPL-treated groups was increased (Figure 4C). These results demonstrate that pretreatment with DPL can suppress constipation.

### 3.4. The Laxative Effect of DPL on Rats with Low-Fiber Diet-Induced Constipation

**Figure 5:**
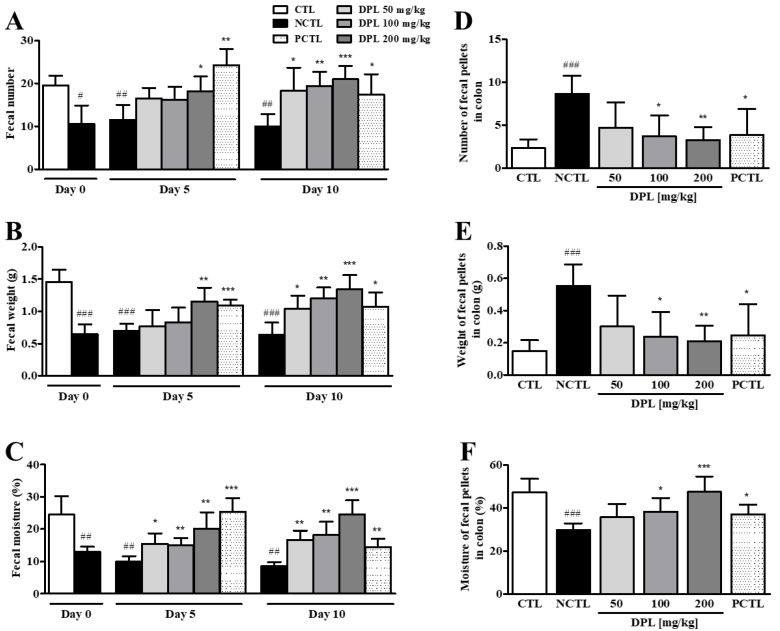
The laxative effects of *D. morbiferus* H. Lév. leaf extract (DPL) on low-fiber diet-induced constipation. At 0, 5, and 10 days, the total number (**A**), weight (**B**), and water content (**C**) of stools were measured. The stool water content was calculated using the fresh and dry weights of stools. At 10 days, the total number (**D**), weight (**E**), and water content (**F**) of stools contained in the intestines were measured. ^#^ Significant difference at *p* < 0.05, ^##^ at *p* < 0.01, and ^###^ at *p* < 0.001 compared with the control group (CTL). * Significant difference at *p* < 0.05, ** at *p* < 0.01 and *** at *p* < 0.001 compared with the low-fiber diet-induced constipation group (negative control group; NCTL).

As shown in Figure 5, the 50, 100, and 200 mg/kg DPL-treated rats showed significant increases (*p* < 0.05, *p* < 0.01, and *p* < 0.001, respectively) in the number of stools compared with the NCTL at 10 days (Figure 5A). The 50, 100, and 200 mg/kg DPL-treated rats showed significant increases (*p* < 0.05, *p* < 0.01, and *p* < 0.001, respectively) in the weight of stools compared with the NCTL at 10 days (Figure 5B). The 50, 100, and 200 mg/kg DPL-treated rats showed significant increases (*p* < 0.01, *p* < 0.01 and *p* < 0.001, respectively) in the water content of stools compared with the NCTL at 10 days (Figure 5C). Bisacodyl was used as the positive control (PCTL), and the bisacodyl-treated group showed significant increases in the number (*p* < 0.05), weight (*p* < 0.05), and water content (*p* < 0.01) of stools compared with the NCTL at 10 days. Fewer feces remained in the colons of rats treated with DPL than in the colons of rats in the NCTL on the last day of the experiment (Figure 5D). The remnant fecal weight in the colon was also dose-dependently decreased in rats treated with DPL (Figure 5E). On the other hand, the 100 and 200 mg/kg DPL-treated groups showed significant increases (*p* < 0.05 and *p* < 0.001, respectively) in the moisture of stools compared with the NCTL (Figure 5F).

### 3.5. The Effect of DPL on Charcoal Meal Gastrointestinal (GI) Motility in Rats with Low-Fiber Diet-Induced Constipation

To evaluate the effects of DPL on the GI tract, we monitored GI motility in the rats. Changes in GI motility by DPL treatment are shown in Table 1 (preventative effects; pretreatment protocol) and Table 2 (therapeutic effects; post-treatment protocol). The time required for the excretion of charcoal meal containing stools in the NCTL (10–24 h) was approximately 4–6 h later than that in the CTL (4–22 h); the number of feces was decreased in the NCTL compared with the CTL. Table 1 presents the results of the preventative test: the 50 and 100 mg/kg DPL-treated groups excreted more feces than the NCTL at 6–10 h, and the 200 mg/kg DPL-treated group showed more rapid fecal excretion than the NCTL. Additionally, a similar pattern was observed in the therapeutic test (Table 2) on GI motility. Taken together, these results demonstrate that DPL treatment can enhance GI motility in the low-fiber diet-induced constipation model.

### 3.6. Effect of DPL Administration on Feeding Behavior in Rats with Loperamide-Induced Constipation

As shown in Supplemental Table S3, the food and water intake were not significantly different among the experimental groups. Similar results were obtained for the body weights of all groups both before and after constipation induction. These results demonstrate that loperamide and DPL administration did not induce alterations in body weight gain, food intake, or water intake under our experimental conditions.

### 3.7. Preventive Effects of DPL Pretreatment on Loperamide-Induced Constipation

**Figure 6:**
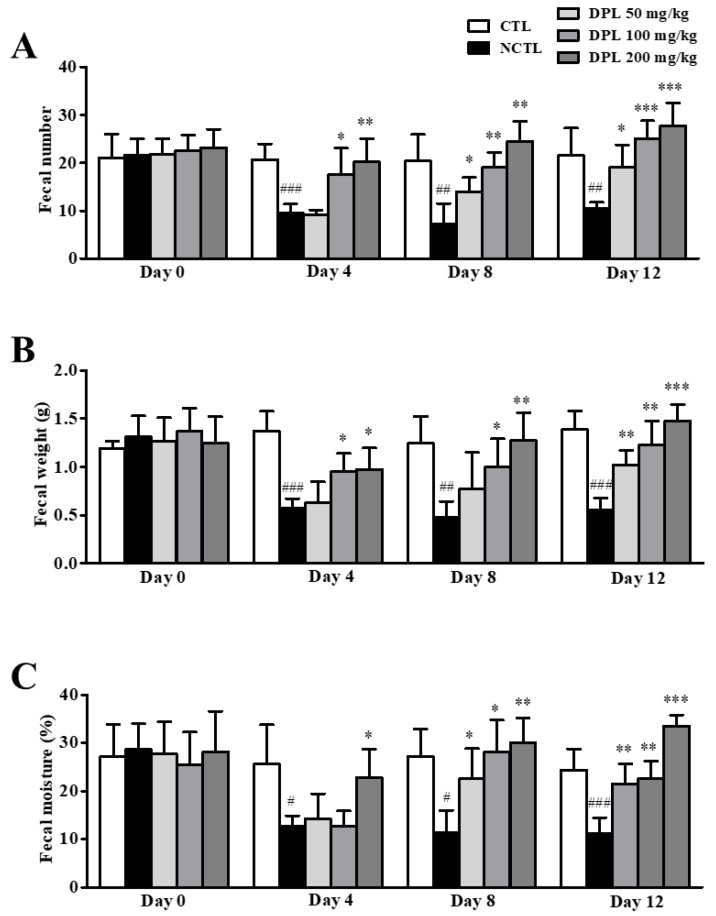
The preventive effects of *D. morbiferus* H. Lév. leaf extract (DPL) on loperamide-induced constipation. At 0, 4, 8, and 12 days, the total number (**A**), weight (**B**), and water content (**C**) of stools were measured. The fecal water content was calculated using the fresh and dry weights of stools. ^#^ Significant difference at *p* < 0.05, ^##^ at *p* < 0.01, and ^###^ at *p* < 0.001 compared with the control group (CTL). * Significant difference at *p* < 0.05, ** at *p* < 0.01 and *** at *p* < 0.001 compared with the loperamide-induced constipation group (negative control group; NCTL).

The fecal parameters before and after loperamide treatment in rats administered DPL are shown in Figure 6. Before loperamide treatment (day 0), the number, weight, and moisture of stools did not significantly differ among the groups. However, after loperamide treatment (day 4), the number and weight of stools increased with DPL concentration. Moisture was significantly higher (*p* < 0.05) in the high-dose DPL (200 mg/kg) treatment group than in the NCTL. In addition, after loperamide treatment (day 12), all fecal parameters in the DPL groups were similar to those in the CTL, in which constipation was not induced.

### 3.8. The Laxative Effects of DPL on Rats with Loperamide-Induced Constipation

**Figure 7:**
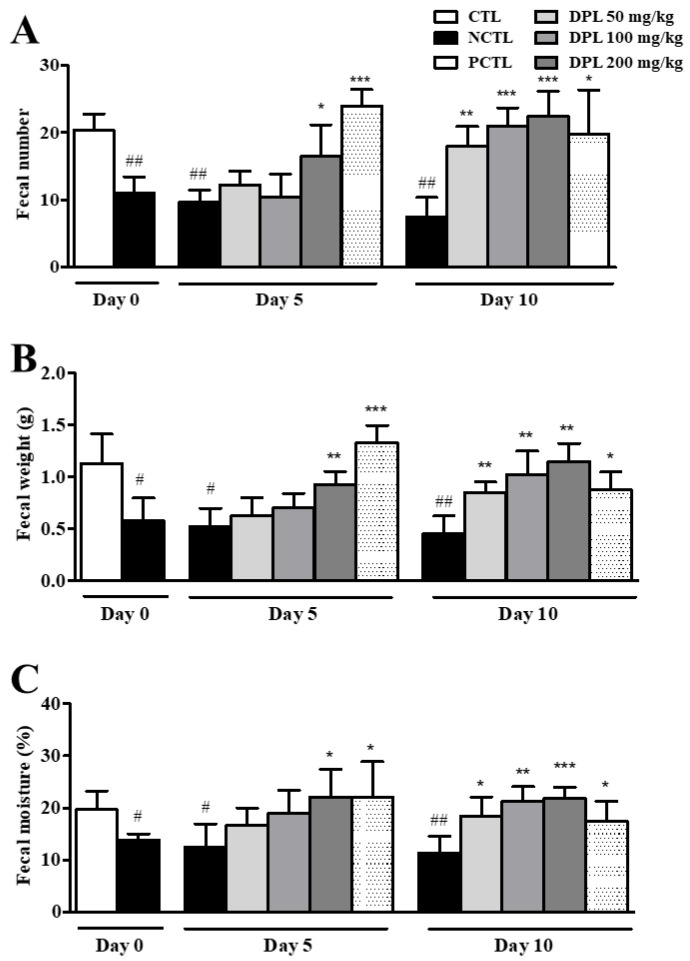
The laxative effects of *D. morbiferus* H. Lév. leaf extract (DPL) on loperamide-induced constipation. At 0, 5, and 10 days, the number (**A**), weight (**B**), and water content (**C**) of stools were measured. The fecal water content was calculated using the fresh and dry weights of stools. ^#^ Significant difference at *p* < 0.05, and ^##^ at *p* < 0.01 compared with the control group (CTL). * Significant difference at *p* < 0.05, ** at *p* < 0.01 and *** at *p* < 0.001 compared with the loperamide-induced constipation group (negative control group; NCTL).

To examine the laxative effect of DPL on the fecal parameters in rats, DPL (50, 100, and 200 mg/kg) was treated once daily for 10 days. As shown in Figure 7, the number (*p* < 0.01), weight (*p* < 0.05), and water content (*p* < 0.05) of stools were decreased after constipation induction (day 0) in all groups, whereas the NCTL showed significant decreases in the fecal number, weight, and water content, rats administered 200 mg/kg DPL showed significant increases in stool number (*p* < 0.05), weight (*p* < 0.01), and water content (*p* < 0.05) starting at 5 days of DPL administration. Furthermore, the oral administration of DPL for 10 days significantly increased the number, weight, and moisture of stools to 18.0 ± 2.94 (*p* < 0.01), 0.85 ± 0.10 g (*p* < 0.01), and 18.46 ± 3.64% (*p* < 0.05) at 50 mg/kg; to 21.0 ± 1.35 (*p* < 0.001), 1.03 ± 0.22 g (*p* < 0.01), and 21.21 ± 2.86% (*p* < 0.01) at 100 mg/kg; and to 22.50 ± 1.85 (*p* < 0.001), 1.15 ± 0.17 g (*p* < 0.01), and 21.82 ± 2.13% (*p* < 0.001) at 200 mg/kg, respectively.

### 3.9. The Effect of DPL on Charcoal Meal Gastrointestinal (GI) Motility in Rats with Loperamide-Induced Constipation

After 10 or 12 days of feeding, charcoal meal was orally administered, and black stool excretion was monitored in each for 24 h (Table 3 and Table 4). Changes in GI motility by DPL treatment are shown in Table 3 (preventative effects; pretreatment protocol) and Table 4 (therapeutic effects; post-treatment protocol). The time required for the excretion of charcoal-containing feces in the loperamide group (10–24 h) was approximately 4–6 h later than that in the control group (6–22 h); the number of feces was decreased in the loperamide group compared with the control group. Table 3 presents the results of the preventative test; the 50 and 100 mg/kg DPL-treated groups excreted more feces than the loperamide group at 8–10 h, and the 200 mg/kg DPL-treated group showed more rapid fecal excretion (6–8 h) than the loperamide group. Additionally, a similar pattern was observed in the therapeutic test (Table 4) on GI motility. Taken together, these results demonstrate that DPL treatment can enhance GI motility in the loperamide-induced constipation rat model.

### 3.10. The Effects of DPL on the PGE_2_ Concentration in RAW264.7 Cells

**Figure 8:**
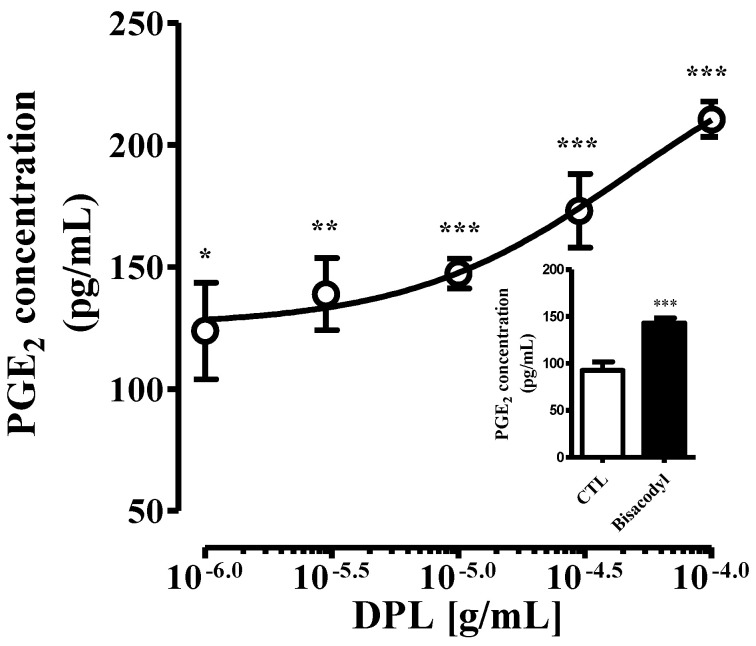
Changes in prostaglandin E2 (PGE_2_) levels in RAW264.7 cells. Thirty minutes after the treatment of *D. morbiferus* H. Lév. leaf extract (DPL), the supernatant was collected, and the levels of PGE_2_ were measured by using enzyme immunoassay (EIA). RAW264.7 cells were administrated with bisacodyl (10 μg/mL) and recovered 30 min later (insert). Each data point represents the mean ± SD of six experiments. * Significant difference at *p* < 0.05, ** at *p* < 0.01, and *** at *p* < 0.001 compared with the control group (control group; CTL).

The main mechanism of bisacodyl, a well-known laxative, is known to directly activate intestinal macrophage to secrete PGE_2_ and regulate the expression of AQP3 in intestinal mucosal epithelial cells by secreted PGE_2_ [24]. Therefore, in this study, it was verified whether DPL, similar to bisacodyl, activates macrophages to regulate the secretion of PGE_2_. The PGE_2_ concentrations in the culture medium 30 min after adding bisacodyl (10 μg/mL) to RAW264.7 cells were significantly increased (*p* < 0.001) compared with those of the control group (Figure 8, insert). Similarly, compared with the control condition, the addition of DPL caused a significant and dose-dependent increase in the PGE_2_ level in the culture medium (Figure 8). We have tested and reported the cytotoxicity of DPL in RAW264.7 cells through former study [13]. We did not observe any cytotoxicity of DPL up to 300 μg/mL in RAW264.7 cells (Supplemental Figure S2).

### 3.11. The Effects of DPL on the PGE_2_ Concentration in the Colons of Rats with Loperamide-Induced Constipation

**Figure 9:**
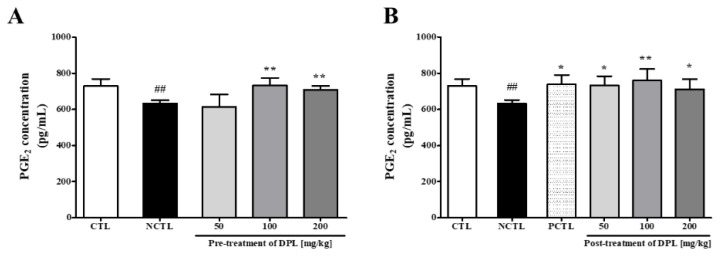
Changes in prostaglandin E2 (PGE_2_) levels in the colon caused by loperamide administration to rats pretreated with *D. morbiferus* H. Lév. leaf extract (DPL) (**A**) and post-treated with DPL (**B**). The concentrations of PGE_2_ were measured by using enzyme immunoassay (EIA). Each bar represents the mean ± SD for seven mice. ^##^ Significant difference at *p* < 0.01 compared with the control group (CTL). * Significant difference at *p* < 0.05 and ** at *p* < 0.01 compared with the loperamide-induced constipation group (negative control group; NCTL).

As shown in Figure 9, PGE_2_ levels in the rat colon were decreased after the induction of constipation with loperamide. PGE_2_ levels were significantly increased in the DPL groups pretreated with 100 and 200 mg/kg compared with the NCTL (Figure 9A). The level of PGE_2_ in the colon was significantly increased (*p* < 0.05, *p* < 0.01, and *p* < 0.05, respectively) in groups post-treated with DPL (50, 100, and 200 mg/kg) compared with the NCTL (Figure 9B).

### 3.12. The Effects of DPL on Colon Contraction

**Figure 10:**
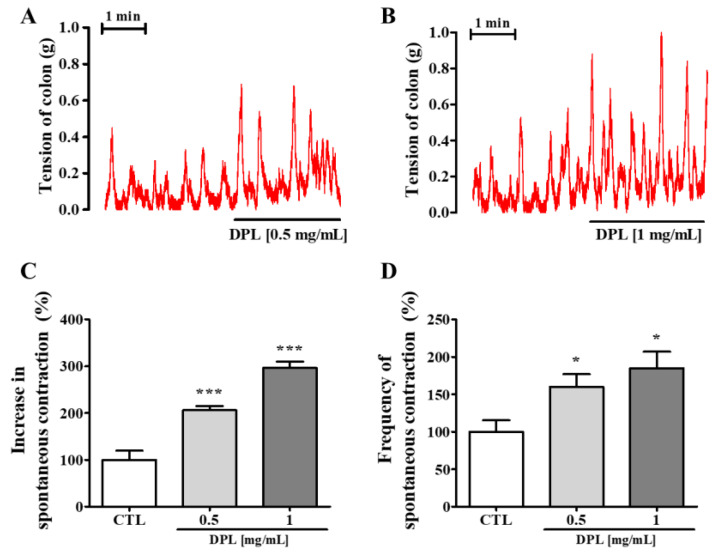
Physiological recordings of spontaneous contractions of the isolated rat colon showing the stimulatory effect of *D. morbiferus* H. Lév. leaf extract (DPL). The stimulation effects of 0.5 mg/mL (**A**) and 1 mg/mL (**B**) DPL. The amplitude of contractions was calculated as the average over 1 min (**C**). The frequency of contractions was measured over 5 min (**D**). Each bar represents the mean ± SD (*n* = 8). * Significant difference at *p* < 0.05 and *** at *p* < 0.001 compared with the baseline (control; CTL).

As shown in Figure 10, DPL induced stimulation immediately after administration and stimulated the rat colon in a dose-dependent manner. As shown in Figure 10C,D, we measured changes in contraction amplitude and frequency by treatment with DPL. The amplitude and frequency of contractions in rat colons were increased by treatment to 0.5 and 1 mg/mL DPL.

### 3.13. The Effects of DPL Fractions on Spontaneous Colon Contraction

**Figure 11:**
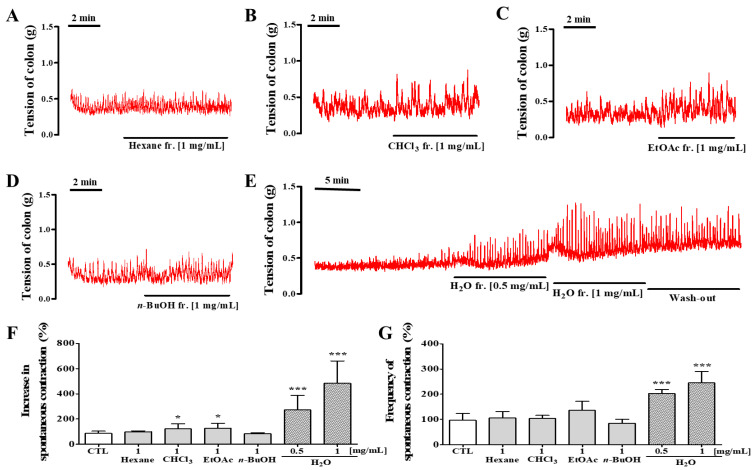
Physiological recordings of spontaneous contractions of the isolated rat colon showing the stimulatory effect of *D. morbiferus* H. Lév. leaf extract (DPL) fractions. The stimulatory effects of (**A**) 1 mg/mL *n*-hexane fraction, (**B**) 1 mg/mL chloroform (CHCl_3_) fraction, (**C**) 1 mg/mL ethyl acetate (EtOAc) fraction, (**D**) 1 mg/mL *n*-butanol (*n*-BuOH) fraction, and (**E**) 0.5–1 mg/mL aqueous (H_2_O) fraction. The amplitude of contractions was calculated as the average over 1 min (**F**). The frequency of contraction was measured over 5 min (**G**). * Significant difference at *p* < 0.05 and *** at *p* < 0.001 compared with the baseline (control; CTL).

We examined the contractive effects of DPL fractions in isolated rat colons (Figure 11). Neither the *n*-hexane fraction nor the *n*-butanol (*n*-BuOH) fraction affected colonic contraction. Compared with the control condition, treatment with the chloroform (CHCl_3_) fraction and ethyl acetate (EtOAc) fraction significantly increased amplitude (*p* < 0.05). However, frequency did not significantly change compared with the CTL. On the other hand, the aqueous (H_2_O) fraction increased not only the contraction amplitude (*p* < 0.001) but also the contractile frequency (*p* < 0.001) in a dose-dependent manner.

## 4. Discussion

Medicinal herbal plants have increased attention as new therapeutics for the treatment of constipation [6]. In the present study, we found that DPL has laxative effects on low-fiber diet- and loperamide-induced constipation model rats because it accelerates the intestinal contraction of rat colons. Furthermore, we investigated the preventive effects and therapeutic effects of DPL on two different constipation rat models, namely rats with low-fiber diet-induced and loperamide-induced constipation. Our results are the first to study how the laxative effects of DPL are strongly related to the promotion of defecation and colon contraction.

The quantitative assessment of natural compounds is helpful for the proper standardization of natural products due to their various pharmacological effects and potential variation. HPLC fingerprints are useful for qualitative and quantitative analysis of natural product formulations. The HPLC chromatograms shown in Figure 2A,B indicate the chromatographic fingerprint of DPL as well the isolation of three organic acids, i.e., neochlorogenic acid, chlorogenic acid, and cryptochlorogenic acid, and two flavonoids, i.e., quercetin and kaempferol, from DPL.

We herein present five principal findings regarding DPL through in vitro, in vivo, and ex vivo studies aimed at explaining the laxative effect of DPL on low-fiber diet-induced and loperamide-induced constipation model rats. First, we found that fecal parameters were increased in the DPL-treated rats compared with the low-fiber diet rats. Fecal excretion is considered an important factor during the development of laxative drugs. An effective laxative should increase the frequency of defecation, reduce stool retention in the colon lumen, and increase the water content of the stool [19,25]. The reduced fecal excretion in low-fiber diet-administered group compared with the normal control group confirmed the low-fiber diet-induced constipation (Figure 3, Figure 4 and Figure 5). Low-fiber diet-induced constipation is related to the water-holding capacity of insoluble dietary fiber [26]. Our results showed that fecal excretion (fecal number, weight, and water content) was significantly increased by the administration of DPL (Figure 3 and Figure 5). Interestingly, we also found significant increases in the fecal moisture and decreases in the fecal numbers and fecal weight in the colons of rats treated with DPL (Figure 4 and Figure 5). Furthermore, after administration of DPL, a significant increase in GI motility was observed, consistent with the bisacodyl treatment (PCTL), which induced similar inhibitory effects on the low-fiber diet-induced decreases in GI motility (Table 1 and Table 2). Taken together, this study demonstrated that DPL prevented and also improved constipation in a rat model of low-fiber diet-induced constipation.

Second, we observed that fecal number, weight, and water content were increased in the DPL-treated rats compared with the loperamide-treated groups. Loperamide is commonly used to produce constipation in animals. Many studies have reported that constipation was successfully induced by administration of 1.5–3 mg/kg loperamide for 3–7 days [19,20,21,22,23]. In the present study, we used loperamide to induce constipation and observed the constipation in animal models administrated with 3 mg/kg loperamide. Furthermore, bisacodyl, and DPL were successfully applied to increase the fecal number, weight, and moisture in a loperamide-induced constipation model (Figure 6 and Figure 7). After administration of DPL, a significant increase in GI motility was observed, consistent with bisacodyl treatment (PCTL), which induced similar inhibitory effects on the loperamide-induced decreases in GI motility (Table 3 and Table 4). GI motility indicated the rapid and mass excretion of feces in the DPL pretreatment rats compared with the low-fiber diet and loperamide rats.

Third, we observed that DPL has a different mechanism from that of bisacodyl and a lower incidence of adverse effects. The oral administration of a bisacodyl to rats increased the fecal moisture [24,25,26,27,28]. Bisacodyl increases the secretion of PGE_2_ in intestinal epithelial cells, which in turn causes the osmotic pressure in the intestinal tract to increase. However, even if the osmotic pressure in the colon increases due to the administration of bisacodyl, the expression level of aquaporin 3 (AQP3) decreases, resulting in a decrease in water movement from the luminal side of the cells [29]. This is because the rate of water movement is more regulated by the expression level of AQPs than by the difference in osmotic pressure [30]. Therefore, long-term administration of bisacodyl can reduce the laxative effect by reducing the expression level of AQP3 [24]. Our results also showed significant increases in fecal number, weight, and water content following the administration of bisacodyl in rat models of low-fiber diet-induced (Figure 5) and loperamide-induced (Figure 7) constipation. However, these effects were shown to decrease when bisacodyl was administered to the rats for a long time (10–12 days). Thus, stimulant laxatives such as bisacodyl are unsuitable for long-term use. On the other hand, the effect of DPL was observed to increase even after treatment for a long period of time, and did not induce tolerance. These results suggest that the laxative effect of DPL may have a different mechanism from that of bisacodyl and a lower incidence of adverse effects.

Fourth, we confirmed that PGE_2_ levels were increased by DPL treatment. PGE_2_ has been implicated in constipation and bacterial infection [31,32,33]. Furthermore, when macrophages are activated, the expression of COX-2 and secretion of PGE_2_ increase [34,35]. Therefore, the effect of DPL on the activation of macrophages was examined using RAW264.7 cells (Figure 8). The concentrations of PGE_2_ in the culture supernatant after the treatment of DPL to RAW264.7 cells were dose-dependently increased. The effect of increasing PGE_2_ secretion by DPL treatment demonstrated in RAW264.7 cells was also verified in loperamide-induce constipation model (Figure 9).

Finally, our results showed that DPL increased not only amplitude of colon contraction but also frequency of colon contraction. The contractions in colons were promoted by treatment to 0.5 and 1 mg/mL DPL (Figure 10). Therefore, we next examined the contractive effects of DPL fractions on isolated rat colons (Figure 11). We verified that the aqueous fraction showed the greatest effect. The better effect of the aqueous fraction compared with the other fractions could be attributed to the type of solvent used in fraction preparation. According to the literature, the aqueous fraction contains mainly chlorogenic acid, and quercetin derivatives [36,37]. However, further studies are needed to determine whether these compounds are involved in the laxative effect of DPL. Many studies have reported that the laxative effect found in plants is due to naringenin, quercetin, terpenoids, phenols, resveratrol, neochlorogenic acid, quercetin, and chlorogenic acid [10,38,39,40]. Among these compounds, quercetin, can effectively improve loperamide-induced constipation animal models [38]. Quercetin was shown to treat constipation by regulating the secretion of mucin [38]. Chlorogenic acid also has the effect of stabilizing intestinal spasms and controlling intestinal inflammation [41]. This research is useful information to advance our understanding of the role of DPL in regulating constipation. Indeed, with the aid of HPLC, a total of five chemicals were validated, including two flavonoids and three organic acids (Figure 2). However, there are reports that quercetin and chlorogenic acid improve colon diseases related to constipation, whereas cryptochlorogenic acid, neochlorogenic acid, and kaempferol have no studies on the relationship with colon diseases. In addition, there are no reports of the pharmacological activity of cryptochlorogenic acid except for one case of anti-inflammatory activity to date [42]. Neochlorogenic acid, a less-studied isomer of chlorogenic acid, has been reported to have antioxidant activity and anticancer effects [43,44]. Kaempferol has been reported to be associated with neuroprotective, anti-inflammatory, antioxidant, and antibacterial activity [45,46]. Therefore, further studies are needed to explore the association between these chemicals and colon disease.

The types of drugs prescribed to patients with chronic constipation can be classified into bulk-forming agents, stool softeners and emollients, osmotic agents, stimulants, chloride channel activators, 5-HT_4_ receptor agonists, and guanylate cyclase-c receptor agonists [47]. The laxative function of DPL demonstrated in this study is closely related to the mechanism of bisacodyl, which is best known as a stimulant. Bisacodyl acts on the myenteric plexus of the colon and stimulates peristaltic contractions, thereby reducing transit time and reducing water absorption from the lumen [47]. However, tolerance was developed with the long-term repeated administration of bisacodyl, whereas no tolerance was observed with the repeated administration of DPL. Therefore, all of these results suggest that DPL has a different laxative mechanism than bisacodyl, and it is necessary to elucidate the detailed mechanism through additional studies. The increased amplitude and frequency induced by DPL and fraction might be mediated by regulating ion channels or voltage-gated Ca^2+^ channels [48,49].

The toxicity of DPL may be one factor involved in intestinal inflammatory or epithelial cell responses. Many researchers have tested and reported the cytotoxicity, acute toxicity, and chronic toxicity of DPL in various cells and animals [13,14,50]. As a result, they have not observed any toxicity of DPL up to 2000 mg/kg/day in 14-day repeated-dose toxicity and 13-week subchronic toxicity study in rats [13]. In addition, we reported that there were no adverse effects in the DPL intake group through other clinical studies [14]. Although this study did not provide direct toxicity results, it is expected that there will be no toxicity according to the results of previous reports. Therefore, in the next study, it is necessary to study the direct effect on the cytotoxicity, acute toxicity, and chronic toxicity of DPL in various cells and animals.

Our studies have demonstrated that DPL, especially the aqueous fraction of DPL, is effective in promoting defecation and colon contractility. Our findings provide, for the first time, scientific evidence of rapid defecation induced by DPL in various constipation animal models and offers potential as a new therapeutic agent for the treatment of constipation.

## 5. Conclusions

This study has shown that DPL has laxative effects on low-fiber diet-induced and loperamide-induced constipation rat models because it accelerates the contraction of rat colons. In addition, the aqueous fraction of DPL effectively stimulated the spontaneous contraction of the colon. In conclusion, these results scientifically support the use of DPL as a natural product-derived laxative without causing diarrhea and tolerance.

## Figures and Tables

**Table 1:** The preventive effects of *D. morbiferus* H. Lév. leaf extract (DPL) on gastrointestinal (GI) motility in rats.

	Mean Number of Charcoal-Containing Stools/2 h (*n* = 7)											
Time (h)	0–2	2–4	4–6	6–8	8–10	10–12	12–14	14–16	16–18	18–20	20–22	22–24
CTL	-	-	0.4	0.7	1.0	2.1	1.4	1.3	1.3	1.7	1.3	-
Low fiber	-	-	-	-	-	1.8	1.5	3	0.8	0.6	0.5	0.2
DPL 50	-	-	-	0.3	1.0	1.2	0.8	2	0.8	1.2	0.5	0.2
DPL 100	-	-	-	0.2	0.7	3	1.0	1.0	1.8	1.3	0.7	0.5
DPL 200	-	-	0.3	0.3	1.2	2.5	0.8	2.2	1.0	1.5	1.3	-

**Table 2:** The therapeutic effects of *D. morbiferus* H. Lév. leaf extract (DPL) on gastrointestinal (GI) motility in rats.

	Mean Number of Charcoal-Containing Stools/2 h (*n* = 7)											
Time (h)	0–2	2–4	4–6	6–8	8–10	10–12	12–14	14–16	16–18	18–20	20–22	22–24
CTL	-	-	-	0.5	0.8	2.7	2.8	2.5	1.5	0.8	1.3	-
Low fiber	-	-	-	-	0.8	2.3	1.3	3	0.5	0.3	0.3	0.5
PCTL	-	-	-	0.6	1.7	1.5	0.2	1.3	1.8	1.2	1.7	1.4
DPL 50	-	-	-	0.2	1.8	1.6	1.4	1.4	1.2	2.8	0.8	-
DPL 100	-	-	-	0.2	0.8	1.4	4.0	1.0	1.6	0.2	0.8	0.2
DPL 200	-	-	0.4	0.6	2.6	0.4	3.8	1.6	2.4	1.8	0.8	-

**Table 3:** The preventive effects of *D. morbiferus* H. Lév. leaf extract (DPL) on gastrointestinal (GI) motility in rats.

	Mean Number of Charcoal-Containing Stools/2 h (*n* = 7)											
Time (h)	0–2	2–4	4–6	6–8	8–10	10–12	12–14	14–16	16–18	18–20	20–22	22–24
CTL	-	-	-	1.4	1.2	2.6	2.4	1.6	1.6	1.0	0.6	-
Loperamide	-	-	-	-	-	0.1	1.3	2.0	1.6	1.5	0.5	1.5
DPL 50	-	-	-	-	0.2	2.1	2.3	1.7	1.2	1.3	0.5	0.7
DPL 100	-	-	-	-	1.2	2.7	2.0	1.8	1.5	1.0	0.8	0.5
DPL 200	-	-	-	0.8	1.2	2.2	2.7	1.5	1.3	1.2	1.0	-

**Table 4:** The therapeutic effects of *D. morbiferus* H. Lév. leaf extract (DPL) on gastrointestinal (GI) motility in rats.

	Mean Number of Charcoal-Containing Stools/2 h (*n* = 7)											
Time (h)	0–2	2–4	4–6	6–8	8–10	10–12	12–14	14–16	16–18	18–20	20–22	22–24
CTL	-	-	-	0.8	2.2	2.6	2.0	1.8	1.6	1.0	0.8	-
Loperamide	-	-	-	-	-	0.6	0.8	3.2	1.2	1.5	0.5	1.0
PCTL	-	-	-	-	0.2	1.4	2.2	1.8	1.2	1.2	1.0	0.2
DPL 50	-	-	-	-	-	0.7	1.3	2.2	2.5	1.8	1.7	1.0
DPL 100	-	-	-	-	0.3	1.7	2.3	2.8	2.3	1.4	0.7	-
DPL 200	-	-	-	0.2	1.8	2.6	2.4	1.9	1.4	1.2	0.6	-

## Data Availability

Data are available from the corresponding author upon specific request.

## Supplementary Materials

The following are available online at https://www.mdpi.com/article/10.3390/medicina57111147/s1, Figure S1: Development of partition procedures for DPL, Figure S2: Cell viability of RAW264.7 cells following different concentrations of DPL or bisacodyl exposure measured by MTT assay, Table S1: Composition of the normal diet and the low-fiber diet, Table S2: Measurement of body weight, feed intake, and water intake in Sprague–Dawley (SD) rats with low-fiber diet-induced constipation, Table S3: Measurement of fecal parameters in rats with loperamide-induced constipation.
