# Supplementary material for: Laxative Effects of a Standardized Extract of Dendropanax morbiferus H. Léveille Leaves on Experimental Constipation in Rats
Source: Medicina (Kaunas). 2021 Oct 22;57(11):1147. doi: 10.3390/medicina57111147 (PMC8619072; doi:10.3390/medicina57111147)
Supplement: Supplementary file 1 [file medicina-57-01147-s001.zip › medicina-1430122-supplementary.pdf]

medicina

MDPI

# Supplemental data

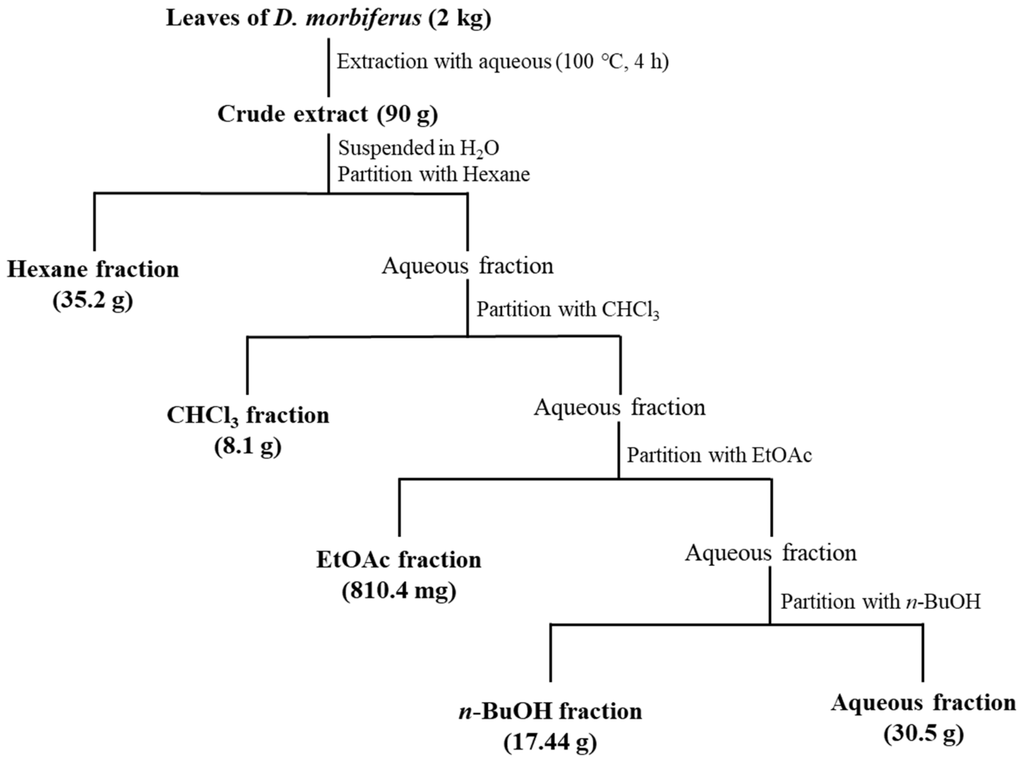


**Supplemental Figure 1.** Development of partition procedures for DPL are summarized in the schematic.

**Supplementary method**

*MTT assay for the measurement of cell viability.*

Cell viability was assessed by a MTT assay. RAW264.7 cells were seeded at a density of 1 × $10^4$ cells/well in a 96-well plate (n=6 replicates) and allowed to adhere overnight. Then, various concentrations of DPL or bisacodyl were treated for 30 min. After the addition of an MTT solution (5mg/ml; 50 $\mu$l/well) and incubation for 4 h, the supernatants were removed and the formazan crystals were solubilized in 100 $\mu$l DMSO. Optical density was determined at 540 nm. Cytotoxicity was expressed as a percentage relative to a control that contained no sample.

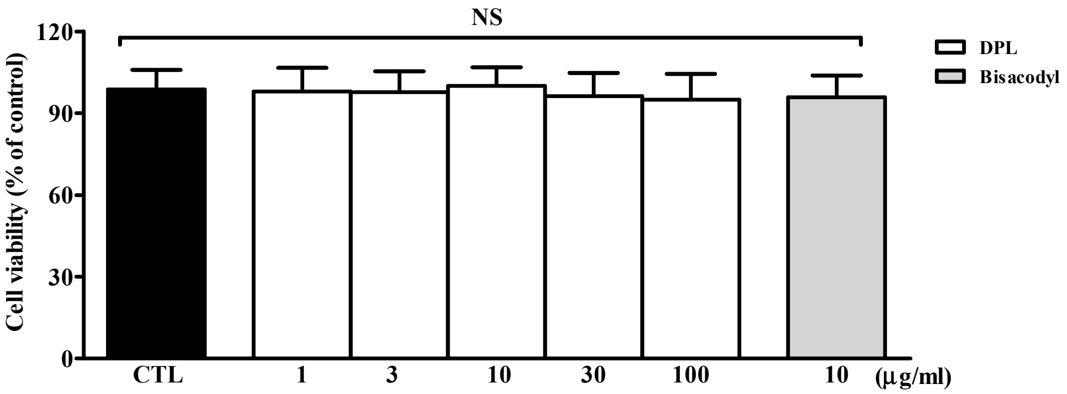


**Supplemental Figure 2.** Cell viability of RAW264.7 cells following different concentrations of DPL or bisacodyl exposure were measured by MTT assay. RAW264.7 cells were exposed to different concentrations of DPL or bisacodyl for 30 min. After exposure, cell viability for each treatment was determined based on spectrometry of formazan formation and represented the viability percentage relative to control exposure. NS; not significant. The data are represented as the means ± SEMs.

**Result:** The cytotoxicity of DPL was evaluated by MTT assay; the viability of cells treated with different concentrations (1, 3, 10, 30 and 100 µg/ml) of DPL compared to control cells was 97.96%, 97.74%, 98.92%, 95.97%, and 93.98%, respectively. Furthermore, the results showed that the viability of the RAW264.7 cells in the presence of bisacodyl at concentrations of 10 µg/ml was not decreased (NS; $P > 0.05$). Therefore, it was decided that the noncytotoxic concentrations of up to 100 $\mu$M of DPL would be used in the following experiments.

**Supplemental Table 1.** Composition of the normal diet and the low-fiber diet.

| Ingredients | Contents (%) | |
|---|---|---|
| | Normal diet | Low-fiber diet |
| Moisture | 9.0 | 9.0 |
| Crude protein | 20.0 | 21.9 |
| Crude fat | 4.5 | 6.1 |
| Crude fiber | 6.0 | 0.1 |
| Crude ash | 7.0 | 5.9 |
| NFE (Nitrogen-free extract) | 53.5 | 57.0 |

**Supplemental Table 2.** Measurement of body weight, feed intake, and water intake in Sprague–Dawley (SD) rats with low-fiber diet-induced constipation. Data are expressed as the mean ± SD.

| | Normal diet group (12 days) | Low-fiber diet group (12 days) | PCTL group (10 days) | Pretreated groups (12 days) | | |
|---|---|---|---|---|---|---|
| | | | | DPL 50 (50 mg/kg) | DPL 100 (100 mg/kg) | DPL 200 (200 mg/kg) |
| | | | | Post-treated groups (10 days) | | |
| | | | | DPL 50 (50 mg/kg) | DPL 100 (100 mg/kg) | DPL 200 (200 mg/kg) |
| **Body weight (g)** | 311.20 ± 26.49 | 301.20 ± 11.61 | 312.75 ± 14.44 | 317.37 ± 18.93 | 315.79 ± 32.75 | 307.68 ± 30.27 |
| | | | | 312.45 ± 19.21 | 305.32 ± 24.78 | 308.59 ± 27.32 |
| **Feed intake (g)** | 18.17 ± 1.96 | 20.43 ± 2.42 | 19.93 ± 2.29 | 21.47 ± 1.80 | 20.66 ± 2.60 | 20.09 ± 4.90 |
| | | | | 20.58 ± 2.76 | 20.36 ± 2.34 | 20.51 ± 2.89 |
| **Water intake (mL)** | 21.60 ± 5.41 | 20.40 ± 3.65 | 22.50 ± 4.50 | 21.05 ± 4.55 | 21.24 ± 5.30 | 20.84 ± 6.67 |
| | | | | 20.11 ± 4.36 | 19.05 ± 4.29 | 20.79 ± 4.14 |

**Supplemental Table 3.** Measurement of fecal parameters in rats with loperamide-induced constipation.

| | Normal diet group (12 days) | Loperamide group (12 days) | PCTL group (10 days) | Pretreated groups (12 days) | | |
|---|---|---|---|---|---|---|
| | | | | DPL 50 (50 mg/kg) | DPL 100 (100 mg/kg) | DPL 200 (200 mg/kg) |
| | | | | Post-treated groups (10 days) | | |
| | | | | DPL 50 (50 mg/kg) | DPL 100 (100 mg/kg) | DPL 200 (200 mg/kg) |
| **Body weight (g)** | 293.40 ± 18.88 | 278.83 ± 22.73 | 282.00 ± 13.40 | 273.83 ± 12.29 | 287.17 ± 18.66 | 290.00 ± 28.17 |
| | | | | 280.83 ± 11.64 | 292.17 ± 19.60 | 273.17 ± 21.37 |
| **Feed intake (g)** | 15.46 ± 2.56 | 17.13 ± 2.68 | 16.52 ± 1.51 | 17.35 ± 2.59 | 15.35 ± 2.08 | 14.83 ± 3.96 |
| | | | | 15.72 ± 1.60 | 14.87 ± 1.59 | 17.27 ± 2.54 |
| **Water intake (mL)** | 35.00 ± 6.12 | 34.50 ± 5.61 | 35.00 ± 6.12 | 34.50 ± 5.61 | 30.17 ± 3.19 | 29.17 ± 4.26 |
| | | | | 32.83 ± 2.48 | 31.83 ± 4.71 | 30.83 ± 3.19 |
